# Supplementary material for: Effects of neoadjuvant stereotactic body radiotherapy plus adebrelimab and chemotherapy for triple-negative breast cancer: A pilot study
Source: eLife. 2023 Dec 22;12:e91737. doi: 10.7554/eLife.91737 (PMC10746137; doi:10.7554/eLife.91737)
Supplement: Figure 2—source code 1. [file elife-91737-fig2-code1.zip › 07-08-2023-RA-eLife-91737/CODEFigure 2.docx]

0=Positive, 1=Negative; Get exact 95% CI for the proportion of positive;

proc freq data = efficacy;

tables orr/ binomial(cl = exact level = "0");

run;

proc freq data = efficacy;

tables pcr/ binomial(cl = exact level = "0");

run;

proc freq data = efficacy;

tables rcb/ binomial(cl = exact level = "0");

run;

data = read_xlsx("figure.xlsx")

ggplot(data = data,aes(id,decrease))+

geom_bar(stat = "identity",aes(fill = RCB))+

scale_x_continuous(position = "top", breaks=c(1:12)) + # x axis position

#scale_fill_brewer(palette = "Blues") +

scale_fill_manual(values=c("orange","yellow","red"))+

geom_point(data = data1,aes(id,type,color= Stage,shape = Stage),size=5)+

scale_color_manual(values = c("#0072BD","#D95319","pink","#7E2F8E","orange"))+

scale_shape_manual(values = c(19,19,19,19,18))+

ylab("Change in tumor volume from baseline (%)")+

scale_y_continuous( breaks=seq(-100,0,20)) +

theme_classic()+

theme(axis.title.x=element_blank(), axis.ticks.x=element_blank())+

geom_hline(aes(yintercept=0), colour="black", linetype="solid",size=0.5)+

geom_hline(aes(yintercept=-30), colour="black", linetype="dashed",size=1.0)
